# Behavioural and Cognitive-Behavioural Treatments of Parasomnias

**DOI:** 10.1155/2015/786928

**Published:** 2015-05-25

**Authors:** Andrea Galbiati, Fabrizio Rinaldi, Enrico Giora, Luigi Ferini-Strambi, Sara Marelli

**Affiliations:** ^1^Department of Clinical Neurosciences and Department of Neurology, Sleep Disorders Center, San Raffaele Scientific Institute, 20127 Milan, Italy; ^2^Faculty of Psychology, Vita-Salute San Raffaele University, 20132 Milan, Italy; ^3^Clinical Neurology, Section for Neuromuscular Diseases and Neuropathies, University Hospital “Spedali Civili”, 25123 Brescia, Italy

## Abstract

Parasomnias are unpleasant or undesirable behaviours or experiences that occur predominantly during or within close proximity to sleep. Pharmacological treatments of parasomnias are available, but their efficacy is established only for few disorders. Furthermore, most of these disorders tend spontaneously to remit with development. Nonpharmacological treatments therefore represent valid therapeutic choices. This paper reviews behavioural and cognitive-behavioural managements employed for parasomnias. Referring to the ICSD-3 nosology we consider, respectively, NREM parasomnias, REM parasomnias, and other parasomnias. Although the efficacy of some of these treatments is proved, in other cases their clinical evidence cannot be provided because of the small size of the samples. Due to the rarity of some parasomnias, further multicentric researches are needed in order to offer a more complete account of behavioural and cognitive-behavioural treatments efficacy.

## 1. Introduction

The term “parasomnia” is derived from the Greek “para” (meaning “alongside of”) and the Latin word “somnus” (meaning “sleep”). Parasomnias are defined as unpleasant or undesirable behaviours or experiences that occur predominantly during the sleep period or within close proximity to sleep. During the last century these phenomena have been carefully studied both polygraphically and clinically, showing that they encompass a wide number of conditions. According to the International Classification of Sleep Disorders (ICSD-3) parasomnias may be categorized according to the sleep phase of origin: Rapid Eye Movement (REM) sleep parasomnias, non-REM (NREM) sleep parasomnias, or miscellaneous, when they arise independently of sleep stage [1].

Since parasomnias are typically benign experiences that tend to resolve spontaneously in the course of development, they are frequently overlooked both by patients and clinicians. Consequently, few studies concern the treatment of these conditions, and most of these works are uncontrolled and based mostly on case reports.

Pharmacological treatments are available for these disorders, but the evidence of their efficacy is variable depending on the disorder. Furthermore, since most of NREM parasomnias are typical of childhood, particular attention must be paid towards pharmacological side effects. Consequently, behavioural and cognitive-behavioural treatments of sleep disorders received a growing attention in the last decades.

Despite the fact that the best known and validated cognitive and behavioural interventions aim to treat insomnia [2, 3], there is also a number of studies concerning parasomnias. Some of these treatments can be applied to a variety of parasomnias [4], as scheduled night awakenings or daytime naps [5, 6], while others are specific to a single disorder, as in the case of imagery rehearsal therapy for nightmares [7].

A systematic account of nonpharmacological treatments efficacy is lacking. This review will therefore cover the nonpharmacological treatments available for parasomnias, taking into account both controlled studies and case reports.

## 2. NREM Parasomnias

NREM parasomnias are classified on the basis of the behaviours displayed by the patient during the episode [1]. The NREM group includes confusional arousals, sleep terrors, and sleepwalking. The general criteria for these disorders of arousal include: (a) recurrent episodes of incomplete awakening, (b) absent or inappropriate responsiveness, (c) limited or no cognition of dream report, and (d) partial or complete amnesia for the episode. The three subtypes have distinct diagnostic codes, and additional criteria are elaborated for each manifestation of partial arousals. In the ICSD-3, sleep-related eating disorder has also been included within the NREM group, because it has many features in common with these disorders [1]. These frequently overlap and it is not unusual for patients to meet the criteria for more than one of these conditions. Some of NREM parasomnias arise in comorbidity with psychiatric disorders, psychotropic usage, and sleep disorders [8]. A detailed anamnesis on medical, psychiatric, and drug usage is therefore needed for a proper evaluation of NREM parasomnias.

There are no large controlled trials assessing treatment efficacy in most of these conditions. Most of what is known comes from small trials or clinical and anecdotal evidence.

### 2.1. Confusional Arousals

Confusional arousals (CA) are characterized by disoriented behaviour or slow mentation during an arousal from NREM sleep [1]. This condition is the consequence of a partial or incomplete awakening, usually out of slow-wave or stage N3 sleep. Therefore, most of the episodes tend to occur at the same time every night and can be predictable. The patient often displays vocalizations with occasional complex behaviours and typically has a poor recall of events the following day. Attempts to awaken the person are often unsuccessful and may be met with vigorous resistance; occasionally the patient can become aggressive and violent. The prevalence ranges from 2.9% in adults to 17.3% in children [9]. Even though this behaviour is typically benign, sometimes the symptoms may persist into adulthood.

#### 2.1.1. Treatment

The first-line treatment in children with CA is reassurance, because the disorder tends to decline spontaneously with age [10]. Patients should also be taught to avoid any precipitating factors, including central nervous system depressants and sleep deprivation [11].

Attarian suggests scheduled or anticipatory awakening as a behavioural technique to prevent these events [12]. This consists in awakening the child 15 to 20 minutes before the usual time of occurrence of CA, in order to alter the sleep state and therefore to abort the event. During the scheduled awakening, the parent should comfort the child [9]. An episode of CA should be allowed to run its course, unless an attempt to leave the bed or to harm the patient occurs, since efforts to restrain the behaviour may lead to aggressiveness [13].

When CA become frequent or do not respond to behavioural therapy, the possibility of a comorbid sleep disorder should be ruled out. Treating the concomitant conditions may reduce the episodes [14].

### 2.2. Sleep Terrors/Night Terrors/Pavor Nocturnus

Sleep terrors (ST), also known as night terrors or pavor nocturnus, are conditions characterized by an unexpected arousal from stage N3, with an abrupt scream and behavioural manifestations of intense fear. They usually have a sudden onset, and the individual displays behaviours of extreme distress, confused verbalizations, autonomic arousal, and increased body movements, sometimes including sleepwalking. The child cannot be consoled or woken and typically has a partial or complete amnesia of the episodes the next day [1]. Prevalence of ST also varies with age from 1% in the elderly to 6.5% in children [15].

#### 2.2.1. Treatment

The first treatment in children with infrequent episodes of ST is reassurance, since they often outgrow it by late adolescence [10]. Similarly to the episodes of CA, any attempt at interrupting the event by blocking, touching, and grabbing will likely lead the patient to react with an aggressive behaviour and may even cause injuries: then it is not recommended. Some authors suggest to wait for the event to run its course and then to guide the child back to bed [12].

The patients should also be educated to avoid sleep deprivation and other precipitants, such as alcohol and drugs [11]. Furthermore, it is important to make the bedroom as safe as possible to minimize the risk of injury, by sleeping on the ground floor, removing obstructions in the bedroom, and closing doors and windows [10, 12].

When the episodes become more frequent or tend to persist into adulthood, treatment is warranted. A wide range of drugs has been suggested, but potential side effects must be a cause for concern, especially in younger patients. Behavioural methods include psychotherapy [16], relaxation therapy [17], and autogenic training or hypnosis [18].

Anticipatory or scheduled awakening can also be used to prevent ST [19]. A study by Lask [20] reported the use of scheduled awakenings in 19 successive children. Parents were instructed to note at what time the episodes happened and subsequently to wake their child fully 10–15 minutes before the terror occurred. The night terrors stopped within a week of starting treatment, and at followup one year later no relapses were reported [20]. This technique is relatively low-risk, but special attention must be paid when the patient is significantly sleep deprived. In this case it is better to increase total sleep time before intervening specifically to treat ST [6].

Kellerman [21] described a behavioural treatment with relaxation therapy in a three-year-old girl with night terrors and acute leukaemia. The treatment, targeted at reducing anxiety related to parental separation and medical procedures, reduced and eventually eliminated the events. In the same study an analogous treatment with a five-year-old boy was reported with similar results [21].

Kales et al. [16] described the effect of psychotherapy in treating adult patients with night terrors. Out of three patients, two completed psychotherapy and showed a complete disappearance or a clear reduction of the episodes; a third patient discontinued therapy and showed no improvement.

Hurwitz et al. [18] reported the use of hypnosis in the treatment of 27 adult patients with sleepwalking and ST. A total of 74% of patients improved after instruction in self-hypnotic exercises.

### 2.3. Sleepwalking/Somnambulism

Somnambulism, or Sleepwalking (SW), is a parasomnia characterized by complex purposeless tasks and wandering episodes of variable duration, arising from stage N2 or N3. Frequently the patients show no memory of the event [1]. Prevalence of SW varies from 4% in adults to 17% in children less than 13 years of age [22].

#### 2.3.1. Treatment

Most cases of SW are benign and resolve spontaneously without treatment when the child grows up. Hence, the first-line treatment is supportive and includes avoiding sleep deprivation and creating a safe environment, giving the sleepwalker quiet guidance back to bed if necessary. It can be also noteworthy to reassure the patient that SW is not linked with underlying psychiatric illness. Treating other apparent predisposing factors and addressing triggering factors is also important. In particular, reversing comorbid sleep disordered breathing conditions often dramatically diminishes nocturnal behaviours, even when patients only have a mild burden of disease [8].

Nevertheless, if symptoms become chronic and the frequency of SW episodes is severe, a targeted intervention is indicated. A number of nonpharmacologic treatments, including anticipatory awakening, psychotherapy, and hypnotherapy have been reported.

Anticipatory awakenings are used in children with NREM parasomnias [23]. This treatment is most appropriate when SW episodes occur at a highly predictable time each night [10] and parents are willing to implement the treatment protocol for at least 1–4 weeks. This consists in waking the patient just prior to the typical onset of an episode. The evidence for the efficacy of scheduled awakenings as a treatment for SW in young children is limited to few published studies [5, 19, 20]. These uncontrolled case reports showed that treated children had no reoccurrence of parasomnia at 6–12 months of followup. Weaknesses of these studies include lack of reliable data on parent monitoring of SW. Although positive results have been reported in children, there are few data concerning the efficacy in adult patients [5, 19].

Behavioural interventions aimed to change the sleep pattern are also suggested: some authors hypothesized that daytime naps could decrease depth of night-time sleep and reduce the number of partial arousals [24, 25]. However, there are no published reports of the effectiveness of this treatment.

Psychotherapy may be helpful for treating SW in adults. A case report of two patients showed that psychotherapy helped developing strategies to cope with the patient's psychological conflicts [26], leading to a better control of the SW episodes. As shown by other works, in fact, psychological factors can play an important role in triggering SW episodes [27–30]. Nevertheless, other studies have shown no effects of psychological treatment on SW [8].

The evidence for hypnotherapy is based mostly on anecdotal data and case reports. In two studies, a significant improvement after more than 6 months of followup was reported [18, 31]. However, other studies failed to confirm these results [18, 32, 33]. Only one randomized blind trial compared active hypnosis to suggestion hypnotherapy in 11 participants and did not show any additional benefit with the active therapy [32].

To our knowledge, randomized controlled trials of nonpharmacological treatments for somnambulism are lacking. Observational evaluations have generally been retrospective and uncontrolled and have relied solely on self-report of improvement by patients as outcome measures.

### 2.4. Sleep-Related Eating Disorder

The ICSD-3 defines sleep-related eating disorder (SRED) as a NREM sleep parasomnia characterized by recurrent episodes of eating, occurring after sleep onset and accompanied by a reduced level of awareness. The sleep-related eating episodes are not linked to daytime eating disturbances such as bulimia nervosa, binge-eating disorder, or anorexia nervosa [1]. The average age of onset is approximately from 22 to 27 years with a mean of nearly 12 to 16 years before clinical presentation. This condition affects females (65%) more than males, and 80% of patients describe a diminished level of consciousness during eating episodes, with a varying degree of amnesia for the events [34, 35].

The distinction between SRED and Nocturnal Eating Syndrome (NES), which is openly recognized as an eating disorder, is still controversial. The main feature that distinguishes these two conditions is the level of awareness during the food intake, which is not impaired in NES [36]. Furthermore, consumption of at least 25% of intake after the evening meal and morning anorexia are typical features of NES [37].

#### 2.4.1. Treatment

In 1993, Schenck et al. [34] concluded that, in comparison with pharmacological treatments which control night eating and induce loss of excessive weight, cognitive behaviour therapies are ineffective for this disorder. Although not being a controlled study, to our knowledge it represents the only research assessing the efficacy of nonpharmacological interventions versus pharmacotherapy.

Since an association between the usage of zolpidem and SRED has been suggested, a specific comprehensive assessment is requested before starting this therapy in patients with insomnia. In fact, zolpidem may cause or increase the frequency of SRED episodes in patients with sleep pathologies that lead to repeated arousals [38, 39].

All the other behavioural therapies studied in scientific literature refer to NES [40] and are therefore beyond the aim of this review.

## 3. REM Parasomnias

As parasomnias are grouped by the sleep phase in which they occur, REM sleep parasomnias can be defined as unpleasant behaviours and experiences arising from REM sleep. Three main parasomnias of this subtype are recognized: nightmare disorder, recurrent isolated sleep paralysis, and REM sleep behaviour disorder (RBD) [1].

### 3.1. Nightmare Disorder

Nightmares are defined as disturbing mental experiences that are able to awake the sleeper from REM sleep. The experience generated internally by the dreamer seems real and vivid and can cause a wide range of negatively toned experiences like anxiety, fear, terror, anger, rage, and embarrassment that result in somatic manifestations like tachycardia, sweating, and tachypnoea. After the arousal, the subject is able to recover the content of the nightmare and is fully alert. The frequency of nightmares represents the disorder itself [1].

In children nightmares are very common, with a frequency of 75%, while in general population lifetime prevalence of nightmare experiences is close to 100% [41]. Regarding nightmare disorder, the prevalence in preadolescent children is 1.8–6% [42], while in adults it is up to 4% [43].

Although reported in strong relation with Posttraumatic Stress Disorder (PTSD), where the presence of nightmare follows a traumatic event, nightmares can occur also in other pathological conditions, such as drug abuse and stressful events and can be considered as normal reactions to acute and chronic combat and operational stress [44]. Nightmares are also present in major depression (MD) and according to Krakow et al., they pose an independent risk to suicidality [45].

Since nightmares are defined by their qualitative and quantitative features (i.e., content and frequency), a detailed evaluation is fundamental, both from treatment and research. The assessment of nightmares is typically performed with specific questionnaires assessing the frequency, distress, and intensity of the episodes [46–48]. Furthermore, of greater importance are logs and diaries that seem more accurate [41, 49].

#### 3.1.1. Treatment

There are six behavioural and cognitive-behavioural techniques for the treatment of nightmares in the literature: imagery rehearsal therapy (IRT); exposure techniques; exposure, relaxation, and rescripting therapy (ERRT); lucid dreaming therapy (LDT); hypnosis; eye movement desensitization and reprocessing (EMDR).

In IRT the patient is asked to modify the plot of the recurring nightmare during wakefulness by verbal or written form, with a new self-made script in which the unpleasant part and/or the ending of the nightmare is replaced with a more pleasant one [50].

Exposure techniques consist in gradually exposing the patient to the source of the negative part of the nightmare during wakefulness and in safe surroundings [51].

In ERRT different types of intervention such as psychoeducation, sleep hygiene, progressive muscle relaxation, and rescripting the nightmare as exposure are combined together [52].

LDT is a restructuring cognitive technique. The patient is taught to become lucid in his nightmare through daily exercises. Consequently, he will be able to perform actions during the nightmare that will modify its storyline [53, 54].

Hypnosis can be also adopted for the treatment of nightmares. Although the authors stated that the data were “very preliminary,” the treatment seemed to be effective [31].

According to the only meta-analysis investigating the size effect [55], all the previous treatments have a moderate effect, and there is no evidence that one is better than one other.

Also EMDR is employed for nightmares treatment. This is an 8-phase approach in which bilateral eye movement, tones, and taps are used to identify and reprocess the targeted disturbed memories and experiences in order to formulate insight and adaptive behaviour in patient with traumatic experiences. To our knowledge, the efficacy of this technique has been tested only in cohorts of PTSD patients [56, 57], with good outcomes for nightmares. In Silver et al. [56] study, EMDR subjects performed better than controls and other intervention groups, treated with relaxation training and biofeedback, in all variables investigating PTSD symptoms including nightmares. Also in a study by Raboni et al. [57] EMDR improved PTSD symptoms and sleep quality. In this case the quality and the frequency of nightmares have not been specifically investigated but they were addressed as a part of sleep quality which effectively improved [58].

At the moment it remains unclear whether nightmare disorder can equally benefit from all these different interventions, independently of the associated conditions (e.g., MD and PTSD).

### 3.2. Recurrent Isolated Sleep Paralysis

Recurrent isolated sleep paralysis can be defined as the persistence of REM sleep, in terms of muscle atonia, into wakefulness. This condition is identified with an inability to speak and to move the limbs and the trunk at sleep onset or upon awakening from sleep, even though the subject maintains a conscious state and a proper recall of the situation. The duration of the episode may vary from seconds to minutes [1]. This disorder may cause relevant consequences, like fear of sleep and anxiety before going to bed. Near 7.6% of the general population experiences at least one episode during lifetime [59].

#### 3.2.1. Treatment

To our knowledge, no controlled studies of behavioural treatment for this condition are present in the literature. However, a reasonable management of this parasomnia is possible. Since sleep paralysis represents a benign and infrequent parasomnia, reassurance is the first-line treatment [60]. Since cultural elements can influence the clinical presentation of sleep paralysis, cultural explanation has to be taken into account [61].

As the frequency of sleep paralysis can be increased by sleep deprivation, a correct sleep hygiene is highly recommended, especially for shift workers and in circadian disorder induced by jet lag [62]. Furthermore, since daytime naps can trigger episodes of sleep paralyses, a proclivity for daytime sleep must be discouraged [63].

### 3.3. REM Sleep Behaviour Disorder

REM sleep behaviour disorder (RBD) is a parasomnia characterized by the absence of sleep atonia during REM sleep and acting out of dreams, leading to injurious or potentially dangerous behaviours to the patients or bedmate [64]. The estimated prevalence is 0.38% in general population and 0.5% in elderly [1]. RBD can occur in a chronic as well as an acute form. Acute forms are connected mainly with withdrawal states from sedative-hypnotic agents and alcohol [65]. It is important to note that some serotonergic antidepressants are known to cause RBD elevating tonic submental electromyogram activity during REM sleep [66]. The chronic form of RBD can be either idiopathic or associated with neurological disorders, most notably synucleinopathies such as Parkinson's disease, multiple system atrophy, and dementia with Lewy body disease [67, 68].

#### 3.3.1. Treatment

RBD represents the only parasomnia for which the pharmacological intervention is the clear mainstay of the treatment. However, controlling environmental safety is a fundamental intervention. Hence, likely dangerous objects should be removed from the bedroom, windows protected, mattresses positioned on the floor, and cushions put around the bed. These interventions are even more important when drug intolerance or ineffectiveness develops [69]. Even though environmental manipulations are suggested as an effective treatment, to our knowledge, RCT studies are still lacking in the literature.

## 4. Other Parasomnias

According to ICSD-3, under the category “other parasomnias” are enlisted all parasomnias that are not related to a specific sleep stage [1].

### 4.1. Exploding Head Syndrome

Exploding head syndrome (EHS) is characterized by the perception of an abrupt, sudden, loud noise or sense of explosion in the head when going to sleep or waking up [70]. These experiences are usually painless, but the patient can be terrified and the episodes result in a great deal of fear, confusion, and distress. Usually, it is a benign and infrequent experience, but it can also flow into a chronic form and result in relevant clinical consequences. Accurate prevalence rates are still lacking since a great part of the literature refers to case studies. EHS is a rare condition [71] and just few studies reported data in small samples [72].

#### 4.1.1. Treatment

No controlled clinical trial is currently reported in the literature for this syndrome. A nonpharmacological treatment is anyway possible and may be effective. In different studies [71, 73, 74] it is suggested that education and reassurance that EHS is a fairly benign condition are helpful. In a case study, Ganguly et al. [71] reported that the reassurance itself led to a remission of the episodes after a 6-month followup. Obviously, further evidence for treatment efficacy is needed.

### 4.2. Sleep-Related Hallucinations

Sleep-related hallucinations are hallucinatory experiences, in particular visual phenomena, that occur at sleep onset (hypnagogic) or on awakening (hypnopompic) [75]. It has been suggested that they can be caused by a REM sleep intrusion into wakefulness, and it may be difficult to differentiate these experiences from dream occurring just prior to waking [76]. A less frequent variation of this form is represented by prolonged, long, vivid visual hallucination after waking during the night. They can occur as isolated phenomena or be a part of the symptomatic picture of narcolepsy. The prevalence of hypnagogic hallucinations is up to 25%, and they are more common than hypnopompic ones that occur in 7% of the general population [77].

#### 4.2.1. Treatment

Being the least studied parasomnia, little is known about treatment possibilities. Different studies suggest that sleep deprivation, cigarette smoking, and certain medications such as *β*-adrenergic agonists, sedative hypnotics, and certain antidepressant can trigger the episodes. Therefore, avoiding these primary causes can relieve the problems [12]. No specific interventions have been evaluated for sleep-related hallucinations. Silber et al. [76] reported two patients treated with hypnosis, although with limited success. It must be underlined that, in healthy individuals, both hypnopompic and hypnagogic hallucinations tend to resolve spontaneously with time.

### 4.3. Enuresis

Sleep enuresis is characterized by the intermittent involuntary voiding during sleep, in absence of a physical disease, in children older than 5 years. Episodes can occur either during REM or NREM sleep [78]. For the diagnosis at least one episode a month for at least three months is required. This condition is more common in children with developmental delay, physical or intellectual disabilities, and psychological or behavioural disorders. It is very common in childhood, with a frequency of almost 10–16% at 5 years, and probably more prevalent in adolescence and adulthood than generally thought [78].

#### 4.3.1. Treatment

A consistent literature about nonpharmacological treatment of nocturnal enuresis is present [79]. Conservative nonpharmacological treatments include education and support about this condition combined with advices about avoiding caffeine based drinks and encouraging adequate fluid intake. The patients must be taught to void every two or three hours during the day and to avoid holding when they feel to urinate. Moreover, instructions about comfortable posture will help the patient to relax pelvic floor muscles [79]. As in several behavioural and psychological disorders, rewarding agreed behaviours (such as drinking adequately, voiding before sleep, and engaging in management) can be more effective than rewarding what is out of the patient's control, that is to say, dry nights [80].

One of the most dated techniques is the alarm training, employed since 1938 [81]. As reported by a systematic review [82] of 56 trials in children, it is the most effective long term treatment. Alarms act as an operant behavioural technique. Children are trained to suppress bladder emptying during sleep or to wake to void by signalling when they urinate, with the usage of bell and pad alarms placed on the bed, whereas personal alarms are worn in the child's underwear. Both types are equally effective [83].

Children with nocturnal enuresis have an increased risk of psychological and behavioural disorders. When marked symptoms are present, a full psychological or psychiatric assessment is recommended. The NICE guideline concludes that there is no evidence to justify the cost of psychotherapy for enuresis without clinically relevant psychological disorder. In this particular case, NICE recommends treating comorbid disorders, in order to improve the adherence to enuresis treatment [80].

Other complementary treatments (i.e., acupuncture) have been used for sleep enuresis. Among them hypnotherapy has been studied in one small randomized controlled trial, in which it appeared to be as effective as a pharmacological intervention (imipramine), with a lower relapse rate after cessation of treatment [84].

## 5. Conclusion

Several behavioural and cognitive-behavioural treatments of parasomnias are available. They have the obvious advantage of avoiding the risk of side effects of pharmacotherapy and deserve particular attention.

As this paper shows, the literature to date is based mainly on case reports or uncontrolled studies in small samples, offering only few examples of well-designed researches. Concerning sleepwalking, nightmares, and enuresis there is a number of controlled trials that allow evaluating the efficacy of these treatments. However, not all these studies of nonpharmacological treatments in sleepwalking were performed with adult patients, and the number of patients enrolled for the other studies was often limited. On the other hand, most of the behavioural and cognitive-behavioural treatments of other parasomnias cannot rely on valuable evidence.

Nonetheless, a nonpharmacologic approach to sleep disorders carries several further benefits. First of all, cognitive and behavioural treatment implies that the patient has an active part in his own treatment. This can possibly lead to a long term benefit to the patient if he learns how to recognize the signs of his disease and how to cope with it. Also, this can have a repercussion on the patient's awareness and ultimately on his quality of life.

Because of the rarity of some parasomnias and the limits of the studies reviewed in this paper, larger multicentric trials would be required. This will help to further evaluate the efficacy of behavioural and cognitive-behavioural treatments of parasomnias in adults and to compare them to pharmacologic treatments.
